# Alterations in the Intestinal Morphology, Gut Microbiota, and Trace Mineral Status Following Intra-Amniotic Administration (*Gallus gallus*) of Teff (*Eragrostis tef*) Seed Extracts

**DOI:** 10.3390/nu12103020

**Published:** 2020-10-02

**Authors:** Johnathon Carboni, Spenser Reed, Nikolai Kolba, Adi Eshel, Omry Koren, Elad Tako

**Affiliations:** 1 Department of Biological Sciences, Cornell University, Ithaca, NY 14853, USA; jrc438@cornell.edu; 2 Department of Food Science, Cornell University, Stocking Hall, Ithaca, NY 14853-7201, USA; smr292@email.arizona.edu (S.R.); nk598@cornell.edu (N.K.); 3 Department of Family Medicine, Kaiser Permanente Fontana Medical Centers, Fontana, CA 92335, USA; 4 Azrieli Faculty of Medicine, Bar-Ilan University, 1311502 Safed, Israel; adizimer@gmail.com (A.E.); omry.koren@biu.ac.il (O.K.)

**Keywords:** teff, staple food crops, prebiotics, probiotics, iron deficiency, zinc deficiency, gut microbiota

## Abstract

The consumption of teff (*Eragrostis tef*), a gluten-free cereal grain, has increased due to its dense nutrient composition including complex carbohydrates, unsaturated fatty acids, trace minerals (especially Fe), and phytochemicals. This study utilized the clinically-validated *Gallus gallus* intra amniotic feeding model to assess the effects of intra-amniotic administration of teff extracts versus controls using seven groups: (1) non-injected; (2) 18Ω H_2_O injected; (3) 5% inulin; (4) teff extract 1%; (5) teff extract 2.5%; (6) teff extract 5%; and (7) teff extract 7.5%. The treatment groups were compared to each other and to controls. Our data demonstrated a significant improvement in hepatic iron (Fe) and zinc (Zn) concentration and LA:DGLA ratio without concomitant serum concentration changes, up-regulation of various Fe and Zn brush border membrane proteins, and beneficial morphological changes to duodenal villi and goblet cells. No significant taxonomic alterations were observed using 16S rRNA sequencing of the cecal microbiota. Several important bacterial metabolic pathways were differentially enriched in the teff group, likely due to teff’s high relative fiber concentration, demonstrating an important bacterial-host interaction that contributed to improvements in the physiological status of Fe and Zn. Therefore, teff appeared to represent a promising staple food crop and should be further evaluated.

## 1. Introduction

Iron (Fe) and zinc (Zn) deficiencies are prevalent public health crises worldwide but especially so in Africa, Latin America, and other parts of the developing world [1,2]. The etiology of these deficiencies includes a lack of substantial meat consumption in combination with a reliance on relatively poor sources of dietary Fe and Zn including grains and cereals. As it pertains to the latter, intrinsic dietary factors that limit Fe and Zn bioavailability such as phytic acid and polyphenolic compounds are often present in increased quantities in staple food crops [3,4,5]. Despite this, other intrinsic dietary factors such as prebiotic-like compounds have the potential to offset the inhibitor-like effects of phytate and polyphenols, thus improving mineral bioavailability [6]. 

Teff (*Eragrostis tef*), a staple cereal grain mainly consumed by peoples of Eritrea and Ethiopia, is gaining notoriety due to its dense nutrient composition including complex carbohydrates, unsaturated fatty acids, trace minerals (especially Fe), and phytochemicals [7,8]. Teff contains high amounts of phytates and polyphenols, although this amount varies by species and is comparable to values reported for other wholegrain cereals. Despite this, a recent study demonstrated that the prevalence of Fe and Zn deficiencies was much lower in Ethiopia relative to other African nations such as Kenya, Nigeria, and South Africa, likely due to the disproportionate dietary intake of teff relative to other cereal grains [9]. Furthermore, as teff is a gluten free cereal grain with high levels of trace minerals, it is well tolerated by individuals with food allergies and other gastrointestinal disorders, such as inflammatory bowel diseases, which negatively impact gut mineral absorption [7].

In addition to its favorable micronutrient profile, the fiber content of teff is uniquely high, especially when compared to other staple food crops such as sorghum, rice, maize, and wheat [7]. Constituents of fiber, such as the prebiotics inulin and raffinose, survive initial digestion in the upper gastrointestinal tract and become subsequently fermented by specific resident commensal bacteria in the colon [10]. This fermentation leads to the production of short-chain fatty acids (SCFA) [11]. SCFAs inhibit the growth of harmful pathogens, decrease intestinal pH, upregulate epithelial cell differentiation, thus increasing villus surface area, and upregulate brush-border membrane (BBM) gene expression [12]. In sum, these effects enhance gut mineral bioavailability and absorption [6,13,14,15,16]. Additionally, it has been suggested that specific polyphenolic compounds found in teff—such as ferulic acid, vanillic acid, cinnamic acid, and coumaric acid—exert a prebiotic effect and may improve trace mineral gut absorption [17].

The consumption of teff continues to rise as demand for well-tolerated, highly nutritious staple food crops continues to increase worldwide, especially as it pertains to biofortification efforts and other population-wide strategies for combating Fe and Zn deficiency [18]. Given the nutritional advantages of teff, especially its high concentration of trace minerals, it is important to evaluate other intrinsic factors that influence absorption and bioavailability. Therefore, using the validated, well-established *Gallus gallus* in vivo model [6,12,13,14,15,19,20], we sought to assess the effects of the intra-amniotic administration of teff seed extracts on serum and tissue Fe and Zn status, as well as on multiple parameters that influence trace mineral absorption and bioavailability, i.e., intrinsic phytate and polyphenolic concentration, enterocyte gene expression of various trace mineral dependent proteins, and brush border membrane morphology. Additionally, 16S rRNA gene sequencing of cecal contents was utilized to analyze potential alterations in intestinal microbiota structure and function from teff extracts. We hypothesize that intra-amniotic administration of teff extracts will indeed exert a prebiotic effect leading to increased serum and tissue Fe and Zn concentrations, favorable alterations in brush border membrane function and morphology, and positively restructure the gut microbiota. 

## 2. Materials and Methods

### 2.1. Sample Preparation

Teff seeds (*Eragrostis tef*), purchased at a local grocer in Ithaca, NY were used in the study (Bob’s Red Mill, Milwaukie, OR, USA). To obtain flour, seeds were ground up in three replicates, using a Kinematica Polymix PX-MFC 90 D analytical mill (Kinematica, Luzern, Switzerland) to a particle size of 50 µm.

### 2.2. Extraction of Prebiotics From Teff

The extraction of prebiotics was performed [6,20,21]. Briefly, the teff flour samples were dissolved in distilled water (50 g/L) (60 °C, 60 min) and then centrifuged at 3000× *g* (4 °C) for 25 min to remove particulate matter. The supernatant was collected and dialyzed (MWCO 12–14 kDa) exhaustively against distilled water for 48 h. The dialysate was collected and then lyophilized to yield a fine off-white powder.

### 2.3. Iron, Zinc, Calcium and Magnesium Analysis

Either a 500 mg sample of teff flour, a 100 mg sample of liver tissue (wet weight), or 50 µL of serum were pre-digested in boro-silicate glass tubes with 3 mL of a concentrated ultra-pure nitric acid and perchloric acid mixture (60:40 *v*/*v*) for 16 h at room temperature. Samples were then placed in a digestion block (Martin Machine, Ivesdale, IL, USA) and heated incrementally over 4 h to a temperature of 120 °C with refluxing. After incubating at 120 °C for 2 h, 2 mL of concentrated ultra-pure nitric acid was subsequently added to each sample before raising the digestion block temperature to 145 °C for an additional 2 h. The temperature of the digestion block was then raised to 190 °C and maintained for at least 10 minutes before samples were allowed to cool at room temperature. Digested samples were re-suspended in 20 mL of ultrapure water prior to analysis using ICP-AES (inductively coupled plasma-atomic emission spectroscopy; Thermo iCAP 6500 Series, Thermo Scientific, Cambridge, United Kingdom) with quality control standards (High Purity Standards, Charleston, SC, USA) following every 10 samples. Yttrium purchased from High Purity Standards (10M67-1) was used as an internal standard. All samples were digested and measured with 0.5 μg/mL of Yttrium (final concentration) to ensure batch-to-batch accuracy and to correct for matrix inference during digestion. Serum LA:DGLA ratio, a novel measure of Zn status, was determined as previously published [21].

### 2.4. Phytate Analysis

A 500 mg sample from teff flour and teff extract were first extracted in 10 mL of 0.66 M hydrochloric acid under constant motion for 16 h at room temperature. A 1 mL aliquot of total extract was collected using a wide bore pipet tip and then centrifuged (16,000× *g*) for 10 min to pellet debris. A 0.5 mL sample of supernatant was then neutralized with 0.5 mL 0.75 M sodium hydroxide and stored at −20 °C until the day of analysis. A phytate/total phosphorous kit (K-PHYT; Megazyme International, Ireland) was used to measure liberated phosphorous by phytase and alkaline phosphatase. Phosphorous was quantified by colorimetric analysis as molybdenum blue with phosphorous standards read at a wavelength of 655 nm against the absorbance of a reagent blank. Total phytate concentrations were calculated with Mega-Calc™ by subtracting free phosphate concentrations in the extracts from the total amount of phosphorous that is exclusively released after enzymatic digestion.

### 2.5. Protein and Fiber Analysis

Protein and fiber analyses were performed as previously published by Wiesinger et al. [22]. Briefly, the total nitrogen concentrations were measured in a 500 mg sample of teff flour or teff extract by the Dumas combustion method at A&L Great Lakes Laboratories (Fort Wayne, IN, USA) in accordance with AOAC method 968.06. Complete methodology is indicated in the supplementary materials. 

### 2.6. Polyphenol Extraction

Next, 5 mL of methanol:water (50:50 *v*/*v*) was added to either 500 mg of teff flour or teff extract, and vortexed for one minute before incubating in a sonication water bath for 20 min at room temperature. Samples were again vortexed and placed on a compact digital Rocker (Labnet International, Inc., Edison, NJ, USA) at room temperature for 60 min before centrifuging at 4000× *g* for 15 min. Supernatants were filtered with a 0.2 μm Teflon™ syringe filter and stored at −20 °C until chemical analysis.

#### Liquid Chromatography-Mass Spectroscopy (LC-MS) Analysis of Polyphenols

Extracts and standards were analyzed as previously published [23]. Briefly, samples were analyzed with an Agilent 1220 Infinity Liquid Chromatograph (LC; Agilent Technologies, Inc., Santa Clara, CA, USA) coupled to an Advion expressionL^®^ compact mass spectrometer (CMS; Advion Inc., Ithaca, NY, USA). Advion Mass Express™ software was used to control the LC and CMS instrumentation and data acquisition. Individual polyphenols were identified and confirmed by comparison of *m*/*z* and LC retention times with authentic standards. Polyphenol standard curves for flavonoids were derived from integrated areas under UV absorption peaks from 5 replications. Standard curves for caffeic acid, ferulic, and protocatechuic acids were constructed from MS ion intensities using 5 replications. The complete methodology is indicated in the supplementary materials. 

### 2.7. Animals and Study Design

Cornish-cross fertile broiler eggs (*n* = 79) were obtained from a commercial hatchery (Moyer’s chicks, Quakertown, PA, USA). The eggs were incubated under optimal conditions at the Cornell University Animal Science poultry farm incubator. All animal protocols were approved by Cornell University Institutional Animal Care and Use committee (ethic approval code: 2007-0129). Prebiotics in powder form were separately diluted in 18 MΩ H_2_O to determine the concentrations necessary to maintain an osmolarity value (OSM) of less than 320 OSM to ensure that the *Gallus gallus* embryos would not be dehydrated upon injection of the solution. At day 17 of embryonic incubation, eggs containing viable embryos were weighed and divided into 7 groups (*n* = 11–15). All treatment groups were assigned eggs of similar weight frequency distribution. Each group was then injected with the specified solution (1 mL per egg) with a 21-gauge needle into the amniotic fluid, which was identified by candling. The 7 groups were assigned as follows: (1) non-injected; (2) 18Ω H_2_O; (3) 5% inulin; (4) teff extract 1%; (5) teff extract 2.5%; (6) teff extract 5%; and (7) teff extract 7.5%. After all eggs were injected, the injection holes were sealed with cellophane tape and the eggs were placed in hatching baskets such that each treatment was equally represented at each incubator location. Immediately after hatch (21 days) and from each treatment group, chicks were euthanized by CO_2_ exposure and their small intestine, blood, pectoral muscle, cecum, and liver were collected.

### 2.8. Blood Analysis and Hb Measurements

Blood was collected using micro-hematocrit heparinized capillary tubes (Fisher Scientific, Waltham, MA, USA). Blood Hb concentrations were determined spectrophotometrically using the QuantiChrom™ Hemoglobin Assay (DIHB-250, BioAssay Systems, Hayward, CA, USA) following the kit manufacturer’s instructions.

### 2.9. Isolation of Total RNA From Duodenum and Liver Tissue Samples

Total RNA was isolated as previously published [23]. Briefly, total RNA was extracted from 30 mg of the proximal duodenal tissue or liver tissue (*n* = 8) using Qiagen RNeasy Mini Kit (RNeasy Mini Kit, Qiagen Inc., Valencia, CA, USA) according to the manufacturer’s protocol. All steps were carried out under RNase free conditions. RNA was quantified by absorbance at A 260/280. Integrity of the 18S ribosomal RNAs was verified by 1.5% agarose gel electrophoresis followed by ethidium bromide staining. DNA contamination was removed using TURBO DNase treatment and removal kit from AMBION (Austin, TX, USA). The complete methodology is indicated in the supplementary materials.

### 2.10. Real-Time Polymerase Chain Reaction (RT-PCR)

RT-PCR was performed as previously published [23]. Briefly, in order to create the cDNA, a 20 µL reverse transcriptase (RT) reaction was completed in a BioRad C1000 touch thermocycler using the Improm-II Reverse Transcriptase Kit (Catalog #A1250; Promega, Madison, WI, USA). The concentration of cDNA obtained was determined by measuring the absorbance at 260 nm and 280 nm using an extinction coefficient of 33 (for single stranded DNA). Genomic DNA contamination was assessed using a real-time RT-PCR assay for the reference of genes samples. The complete methodology is indicated in the supplementary materials. 

#### 2.10.1. Primer Design

Primer design was conducted as previously published [13] and as indicated in the supplementary materials. The sequences and the description of primers used in this study are summarized in Table 1.

#### 2.10.2. Real-Time qPCR Design

All procedures were conducted as previously described [23]. Briefly, cDNA was used for each 10 µL reaction together with 2×BioRad SSO Advanced Universal SYBR Green Supermix (Cat #1725274, Hercules, CA, USA), which included buffer, Taq DNA polymerase, dNTPs, and SYBR green dye. The data on the expression levels of the genes were obtained as Cp values based on the “second derivative maximum” (automated method) as computed by Bio-Rad CFX Maestro 1.1 (Version 4.1.2433.1219, Hercules, CA, USA). The specificity of the amplified real-time RT-PCR products were verified by a melting curve analysis (60–95 °C) after 40 cycles, which should result in a number of different specific products, each with a specific melting temperature. The complete methodology is indicated in the supplementary materials. 

### 2.11. Collection of Microbial Samples and Intestinal Contents DNA Isolation

The ceca were sterilely removed and contents were treated as described previously [23]. A full description of the method is indicated in the supplementary materials. 

#### 16S rRNA Gene Amplification, Sequencing and Analysis

16S rRNA gene amplification, sequencing, and analysis was performed as previously described [11]. Briefly, microbial genomic DNA was extracted from cecal samples using the PowerSoil DNA isolation kit, as described by the manufacturer (MoBio Laboratories Ltd., Carlsbad, CA, USA). Bacterial 16S rRNA gene sequences were PCR-amplified from each sample using the 515F-806R primers for the V4 hypervariable region of the 16S rRNA gene, including 12-base barcodes. The complete methodology is indicated in the supplementary materials. 

### 2.12. Glycogen Analysis

Glycogen analysis was obtained from pectoralis muscle as previously described [8,23]. Briefly, the pectoral muscle samples were then homogenized in 8% perchloric acid. Samples were then centrifuged at 12,000× *g* at 4 °C for 15 min. The supernatant was removed and 1.0 mL of petroleum ether was added to each tube. After mixing, the petroleum ether fraction was removed and samples from the bottom layer were transferred to a new tube containing 300 µL of color reagent. All samples were read at a wavelength of 450 nm in ELISA reader and the amount of glycogen was calculated according to a standard curve. The amount of glycogen present in pectoral sample was determined by multiplying the weight of the tissue by the amount of glycogen per 1 g of wet tissue. A full description of the method is described in the supplementary materials.

### 2.13. Liver Ferritin Analysis

Liver ferritin analysis was conducted as previously described [8,23]. Briefly, 1 g of sample was diluted into 1 mL of 50 mM Hepes buffer, pH 7.4, and homogenized on ice for 2 min (5000× *g*). One mL of each homogenate was subjected to heat treatment for 10 min at 75 °C to aid isolation of ferritin (other proteins are not stable at that temperature). Subsequently, samples were immediately cooled down on ice for 30 min. Thereafter, samples were centrifuged for 30 min (13,000× *g*) at 4 °C until a clear supernatant was obtained and the pellet containing most of the insoluble denatured proteins was discarded. Native polyacrylamide gel electrophoresis was conducted using a 6% separating gel and a 5% stacking gel. Samples were run at a constant voltage of 100 V. Thereafter, gels were treated with either of the two stains: Coomasie blue G-250 stain, specific for proteins, or potassium ferricyanide (K_3_Fe (CN)_6_) stain, specific for Fe. The corresponding band found in the protein and Fe stained gel was considered to be ferritin. Measurements of the bands were conducted using the Quantity-One-1-D analysis program (Bio-Rad, Hercules, CA, USA). A full description of the method is described in the supplementary materials.

### 2.14. Tissue Morphology Examination

Villus epithelium analysis was performed as was previously described [23]. Samples were fixed in fresh 4% (*v*/*v*) buffered formaldehyde, dehydrated, cleared, and embedded in paraffin. Serial sections were cut at 5 µm and placed on glass slides. Intestinal sections were deparaffinized in xylene, rehydrated in a graded alcohol series, stained with Alcian Blue/Periodic acid-Schiff, and examined by light microscopy. The following variables were measured in the intestine: villus height, villus width, depth of crypts, paneth cells, goblet cell number, goblet cell diameter, types of goblet cells in the villi epithelium, goblet cells within the crypts, and the mucus layer thickness in each segment were performed with a light microscope using EPIX XCAP software (Standard version, Olympus, Waltham, MA, USA). The complete methodology is indicated in the supplementary materials.

### 2.15. Statistical Analysis

All values are expressed as means and standard deviation. Experimental treatments for the intra amniotic administration assay were arranged in a completely randomized design. The results were analyzed by ANOVA. For significant *p*-values, a post-hoc Duncan test was used to compare test groups. Statistical analysis was carried out using SPSS version 20.0 software. The level of significance was established at *p* < 0.05. For the microbiome results, Faith’s Phylogenetic Diversity [24] was used to calculate bacterial richness within each sample. Differences between groups were analyzed by ANOVA. Beta diversity (between samples) was calculated using Jaccard distances and analyzed using a pairwise PERMANOVA test. Predictive metagenomic analysis (PICRUST) [25] was used to identify significant differences in predicted metabolic pathways between the groups. Statistically significant p-values associated with microbial clades and functions identified by LEfSe [26] were corrected for multiple comparisons using the Benjamini–Hochberg false discovery rate (FDR) correction. 

## 3. Results

### 3.1. Concentration of Calcium, Iron, Magnesium, and Zinc in Teff Flour and in Teff Flour Extract

Fe and Zn concentrations were higher in the teff extract compared to the teff seed (*p* < 0.05, Table 2), whereas Ca and Mg concentrations were higher in the teff seed compared to the teff extract (*p* < 0.05, Table 2). The soluble fiber and total fiber content were higher in the teff extract compared to the teff seed, however the insoluble fiber content was higher in the teff seed compared to the teff extract (*p* < 0.05, Table 3). There were no significant differences in protein or phytic acid between the two groups. However, the content of phytate:Fe ratio was significantly greater in the teff seed relative to teff extract (*p* < 0.05).

### 3.2. Polyphenol Profile of the Teff Seed Flour and Extract

The concentration of the five most prevalent polyphenolic compounds found in the teff variety are presented in Table 4. Teff seeds contained high levels of ferulic acid.

### 3.3. Hemoglobin, Body Weight, Cecum Weight, and Cecum:Body Weight Ratio

There were no significant differences in hemoglobin concentrations between any of the treatment groups. The body weight of the non-injected group was significantly lower than all other groups (*p* < 0.05, Table 5). Among cecum weights and cecum:body weight ratio, the non-injected and 2.5% teff groups demonstrated significantly lower values when compared to all other groups (*p* < 0.05).

### 3.4. Hepatic, Serum Fe, and Zn Concentrations: LA:DGLA Ratio

**Figure 1:**
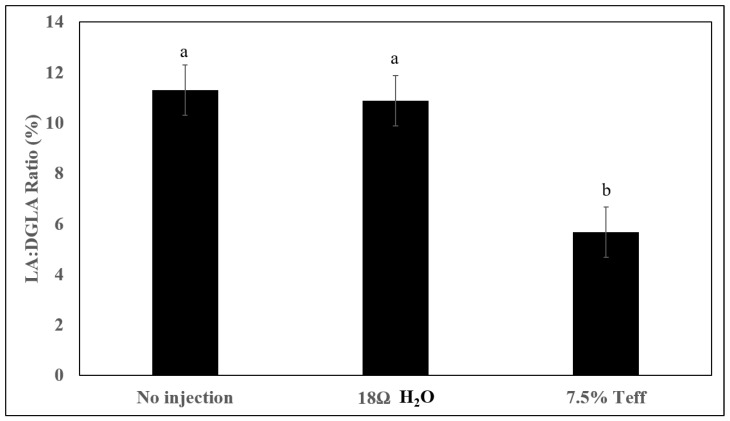
Effect of the intra-amniotic administration of experimental solutions and control on the Linoleic acid (LA): Dihomo-γ-linolenic acid (DGLA) ratio. Values are the means ± SEM, *n* = 8. ^a,b^ Treatment groups not indicated by the same letter are significantly different (*p* < 0.05).

Liver Fe concentration was significantly higher in the inulin and 7.5% teff group compared to the non-injected control group (*p* < 0.05, Table 6). The 18ΩH_2_O, 1% teff, 2.5% teff, and 5% teff groups had higher liver Fe concentration than the control group, although they were not significantly different from each other. The concentration of Zn in the liver was the highest in the 7.5% teff group (similar to the 5% inulin group), and significantly different from all other groups (*p* < 0.05). Serum Fe and Zn concentrations did not different amongst groups (*p* > 0.05). Figure 1 depicts the LA:DGLA ratio for the NI, 18ΩH_2_O, and 7.5% teff groups. The 7.5% teff group demonstrated a significantly lower LA:DGLA ratio, signifying a relative increase in Zn status, compared to both control groups (*p* < 0.05). We have previously demonstrated the LA:DGLA ratio as a novel physiological biomarker of zinc status [19,21]. 

### 3.5. Pectoral Muscle Glycogen Concentration

There were no significant differences in glycogen concentration observed between any of the treatment and control groups (*p* > 0.05, Table 7).

### 3.6. Liver Ferritin Concentration

The non-injected, water, and 1% teff groups had significantly greater levels of liver ferritin compared to all other treatment groups (*p* < 0.05, Table 7).

### 3.7. Duodenal Gene Expression of Relevant Proteins

**Figure 2:**
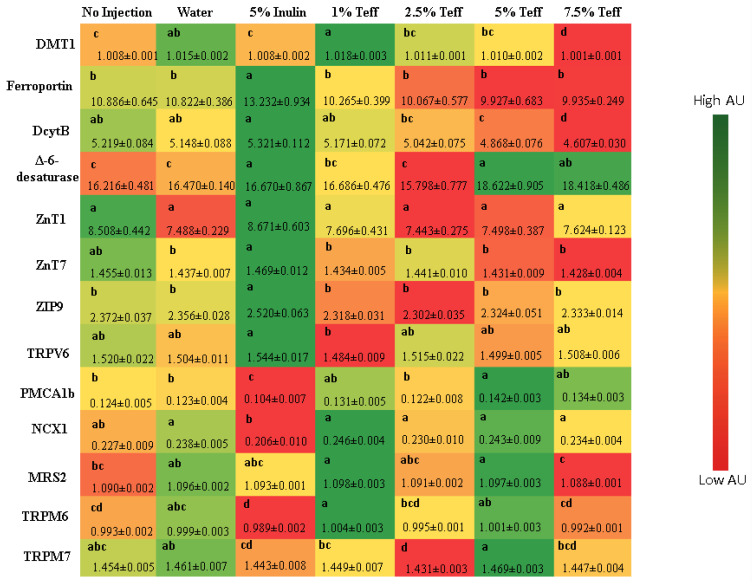
Effect of the intra-amniotic administration of experimental solutions on duodenal gene expression. Values are the means (AU: arbitrary units) ± SEM, *n* = 8. a–d Per gene, treatments groups not indicated by the same letter are significantly different (*p* < 0.05). DMT1, Divalent metal transporter 1; DcytB, Duodenal cytochrome b; ZnT1, Zinc transporter 1; ZnT7, Zinc transporter 7; ZIP9, Zinc transporter 9; TRPV6, Transient Receptor Potential Cation Channel Subfamily V Member 6; PMCA1b, Plasma Membrane Calcium ATP-pump; NCX1, Sodium Calcium Exchanger 1; MRS2, Magnesium transporter MRS2; TRPM6, Transient Receptor Potential Cation Channel Subfamily M member 6; TRPM7, Transient Receptor Potential Cation Channel Subfamily M member 7.

Figure 2 depicts the gene expression of various proteins involved either directly involved in mineral metabolism or indirectly involved requiring these trace minerals as cofactors.

#### 3.7.1. Fe-Related Proteins

For the proteins responsible directly for Fe uptake at the brush border membrane (BBM), ferroportin, and DMT1, the expression was higher in the 5% inulin group and 1% teff group, respectively. The expression of these proteins was lower in the animals receiving the higher teff concentrations. The expression of DctyB was also higher in the 5% inulin group, and decrease in a dose-dependent fashion in the groups receiving increasing teff concentration. 

#### 3.7.2. Zn-Related Proteins

The expression of proteins related to cellular Zn uptake, transport and storage—ZnT1, ZnT7, and ZIP9—was greatest in the non-injected and 5% inulin groups and was significantly lower in all concentrations of teff groups.

#### 3.7.3. Ca-Related Proteins

The expression of proteins related to cellular Ca regulation—TRPV6, PMCA1b, and NXC1—were differentially expressed across groups. TRPV6, a BBM protein involved in the initial steps of Ca absorption in the gut, was highest in the 5% inulin group. The Ca efflux proteins, PMCA1b and NXC1, were expressed highest in the 5% teff group.

#### 3.7.4. Mg-Related Proteins

The expression of the cellular membrane Mg influx proteins TRPM6 and TRPM7 was increased in the 1% teff and 5% teff groups, respectively. MRS2, a Mg-specific protein in the cellular mitochondria, was increased in both 1% and 5% teff groups.

#### 3.7.5. Inflammatory Cytokines and BBM Proteins 

**Figure 3:**
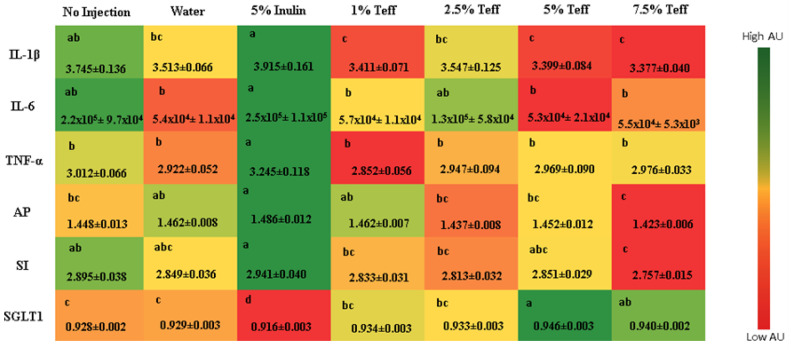
Effect of the intra-amniotic administration of experimental solutions on intestinal and heart gene expression. Values are the means (AU: arbitrary units) ± SEM, *n* = 8. a–c Per gene, treatments groups not indicated by the same letter are significantly different (*p* < 0.05). IL-1β, Interleukin 1 beta; IL-6, Interleukin 6; TNF-α, Tumor Necrosis Factor Alpha; AP, Amino peptidase; SGLT1, Sodium-Glucose transport protein 1; SI, Sucrose isomaltase.

Figure 3 depicts a panel of inflammatory cytokines and BBM proteins evaluated amongst the treatment groups. As a whole, the 5% inulin group had greater expression of all three cytokines relative to the control group, while a decreased expression was seen in the higher concentration teff groups (*p* < 0.05). Both aminopeptidase and sucrose isomaltase were expressed at a higher amount in the 5% inulin group. Likewise, the highest concentration teff group (7.5%) showed the lowest expression. For sodium-glucose transport protein 1, the 5% teff group had the highest expression, with the lowest expression observed in the 5% inulin group.

### 3.8. Morphometric Analysis of Duodenal Villi, Depth of Crypts and Goblet Cells

Table 8 depicts the morphometric measurements for villi length and diameter, crypt depth, and mucus layer width. As a whole, the teff groups showed the most significant response with the 2.5% and 7.5% teff groups with the longest villus length, the 1% teff group with the greatest villus diameter, and the 1% teff group had the greatest intestinal crypt depth. 

Table 9 depicts the morphometric measurements of duodenal goblet cells. As above, the teff treatment groups demonstrated greatest benefit with the 2.5% teff group showing greatest goblet cell diameter, 1% and 2.5% teff groups showing greatest crypt goblet cell count per unit area, and 1% teff group with the greatest villus goblet cell count per unit area (*p* < 0.05). The NI and 5% inulin groups had significantly greater numbers of acidic villus goblet cells than did all other groups (*p* < 0.05). The quantity of neutral goblet cells was significantly greater in the NI group. However, the 2.5% teff group had the greatest amount of mixed villus goblet cell types (*p* < 0.05). 

### 3.9. Analysis of the Gut Microbiota

**Figure 4:**
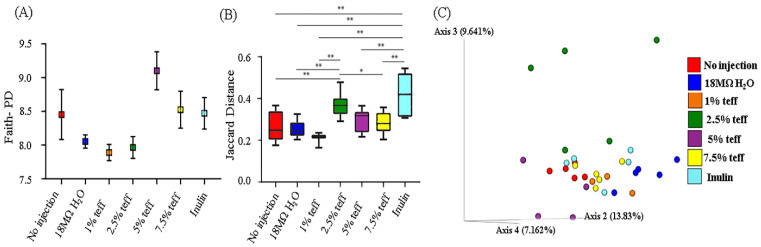
Microbial diversity of the cecal microbiome. (**A**) Measure of α- diversity using Faith’s Phylogenetic Diversity; (**B**) Box-plots of Jaccard distances within the different groups; (**C**) Principal Coordinates Analysis (PCoA) based on Jaccard distances. Each dot represents one animal, and the colors represent the different treatment groups. *n* = 5, * = *p* < 0.05, ** = *p* < 0.01.

Figure 4 represents the observed differences in gut microbial diversity among treatment groups. No significant differences were found in α-diversity using Faith’s phylogenetic diversity (PD) among treatment groups (Figure 4A, *p* > 0.05). Variation between samples (β-diversity) was calculated by using Jaccard distances. The chick microbiotas in the Inulin treatment group were the least similar (biggest distances between samples) to one another compared to the other groups (Figure 4B,C, *p* < 0.01). The distances within the teff treatment groups, except 2.5%, were not significantly different from both the NI control and 18Ω H_2_O groups, which indicates the constant influence of the treatment on the microbial population.

**Figure 5:**
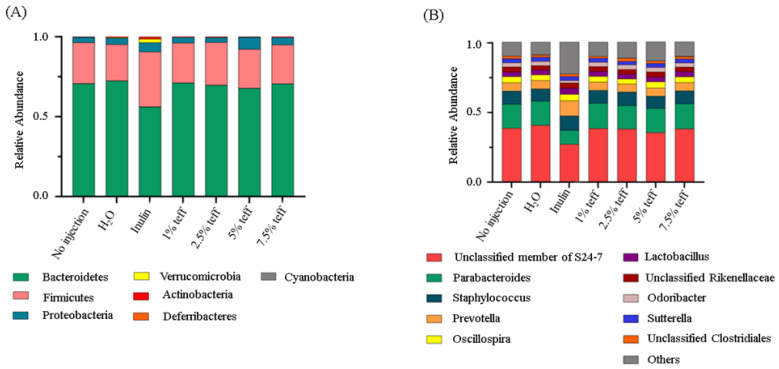
Compositional changes of the gut microbiota. (**A**) Phylum-level differences between the intra-amniotic administration measured at day of hatch (day 21); (**B**) Genus-level differences between the intra-amniotic administration groups as measure at day of hatch (day 21).

Although there were observed cecal microbiota shifts at the phylum and genera level, these differences were not significant (Figure 5, *p* > 0.05). 

**Figure 6:**
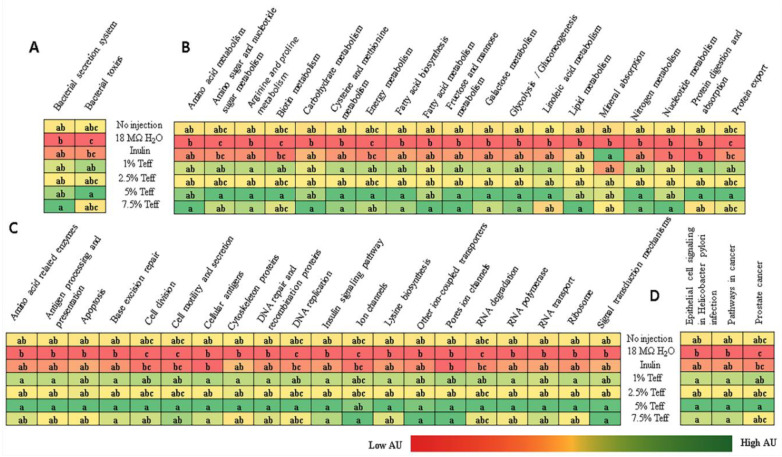
Predictive functional capacity of the cecal microbiota collected at hatch. (**A**) Bacterial processes; (**B**) metabolic processes; (**C**) cellular processes; and (**D**) human diseases. Treatment groups are indicated by their names and groups not indicated by the same letter are significantly different (*p* < 0.05).

We also analyzed whether the genetic capacity of the microbiota could be affected by experimental teff solutions. Metagenome functional predictive analysis was carried out using PICRUSt software, feature abundance was normalized by 16S rRNA gene copy number, identified using the Greengenes database, and Kyoto Encyclopedia of Genes and Genomes (KEGG) orthologs prediction was calculated. Differentially-expressed pathways are displayed in Figure 6. In all but one bacterial metabolic pathway, mineral absorption and relative abundance were greater in teff treatment groups when compared to both NI control and 18Ω H_2_O groups. Bacterial mineral absorption pathway was upregulated in the inulin group when compared to controls and teff treatment groups.

## 4. Discussion

The implementation of teff as an advantageous staple food crop is rapidly growing due to its relative ease of cultivation, sensory quality of teff-based products, and favorable macro- and micronutrient composition [3,18,27]. Compared to other crops, teff does contain notable antinutrients, such as a higher concentration of phytic acid; however, processing methods such as fermentation can drastically lower phytic acid concentration and thus mineral inaccessibility. Despite this, the total polyphenolic content of many teff verities is lower than other food crops including sorghum and cowpea, while specific polyphenols have been shown to aid in mineral bioavailability, including ferulic, caffeic, and protocatechuic acids, which have been found in relatively higher quantities [4,7,8]. Polyphenolic analysis of the teff samples used in this study corroborated previous data demonstrating presence of ferulic, caffeic, and protocatechuic acids. The prevailing wisdom has been that phytate and polyphenolic compounds as a whole disproportionately limit trace mineral bioavailability despite the presence of other promoter-like compounds, such as prebiotics, which can counteract this effect [28,29,30]. We suggest a revised approach to this notion given the favorable physiologic and nutritional benefits afforded by intra-amniotic teff extract administration despite higher phytate concentration.

We have evaluated the effect of intra-amniotic teff extract administration on mineral status and other pertinent physiologic parameters. To our knowledge, ours is the first study to evaluate the effect of teff seed extract on mineral status, duodenal brush border membrane development, and functionality, as well as cecal microbiota. Intra-amniotic teff extract administration increased hepatic Fe and Zn concentrations, although serum Fe and Zn concentrations were not affected. Gene expression analysis demonstrated that teff extract up-regulated certain duodenal mineral transporters, such as DMT1 (BBM major Fe transporter) and ferroportin (BLM major Fe exporter), relative to control. Additionally, hepatic ferritin, the animal’s primary form of cellular Fe storage, was decreased in all teff treatment groups except the 1% concentration, which could be explained by the prioritization of developing an embryo for hemoglobin synthesis versus storage. Indeed, previous intra-amniotic feeding trials have been unable to demonstrate a significant difference in Fe and Zn status, which are secondary to the relatively short treatment exposure time [6,13,23] that carries over to results for serum mineral status. However, the LA:DGLA ratio, a sensitive and specific biomarker of Zn status, was significantly lower in the higher concentration teff extract group demonstrating relative Zn repletion in this group [21,31]. Therefore, it appears that despite the short treatment exposure time, the significant increases in the hepatic Fe and Zn concentrations combined with lower LA:DGLA ratio in the 7.5% concentration group suggest that teff has potentially greater influence on mineral status than other intra amniotic nutritional solutions and purified extracts we have previously tested and thus may demonstrate superiority in delivering bioavailable Fe and Zn [13,32]. 

Further, the duodenal morphometric analysis demonstrated a significant (*p* < 0.05) effect on villus length, diameter, and crypt depth in all experimental teff groups. This effect was not concentration-dependent. Additionally, a significant (*p* < 0.05) increase in neutral and mixed duodenal goblet cells was observed in all teff groups when compared to both water-injected and non-injected controls. Increased number and size of duodenal goblet cells is a surrogate for increased luminal mucin production and secretion, which serves as a defensive barrier to pathogens and promotes epithelial cell function. Additionally, an improvement in villus architecture yields an increased surface area, thus improving the digestive enzyme and absorptive capacity [21,33,34]. The brush border gene expression analysis was mixed, with teff groups showing upregulated SGLT-1 expression while the inulin group showing a relative increase in SI and AP expression. Taken as a whole, this data demonstrates that the intra-amniotic administration of teff extract can modulate brush border membrane development and functionality. These results are in accordance with other intra amniotic administration trials that used isolated prebiotic polysaccharide compounds [12,13,14,15,32]. 

Together with beneficial changes in physiological parameters and brush border membrane functionality occurring as a result of intra-amniotic administration of teff extracts, we found low inter-individual variation among the different teff treatment groups (except of 2.5%) compared to the Inulin treatment. Significant differences in the composition of the cecal microbiotas were not observed. Using KEGG analysis, for the exception of bacterial mineral absorption, the functional capacity of the cecal microbiota demonstrated up-regulation in all bacterial metabolic pathways in the higher concentration teff groups. Given that brush border absorptive capacity was significantly increased in the teff groups, lower bacterial mineral metabolism in the teff group was likely due to less free Fe and Zn present in cecal contents for bacteria to utilize, and hence a compensatory upregulation in the bacterial mineral absorption pathway. 

Several notable pathways upregulated in the teff groups included bacterial energy metabolism, sugar/carbohydrate metabolism, fatty acid biosynthesis, and protein synthesis. This was likely due, in part, to the large fiber content present in teff [7]. Indeed, relative to other staple food crops such as wheat, sorghum, and maize, teff contains several folds higher concentration of crude fiber, total, and soluble dietary fiber [7]. Fiber delivered to cecal bacteria exerts a prebiotic-like effect by allowing for the proliferation of butyrate- and other SCFA-producing bacteria. We demonstrated that bacterial fatty acid production was significantly increased in the teff extract groups. SCFAs have been shown to promote the proliferation and differentiation of intestinal mucosal epithelial cells [35,36]. Additionally, in the presence of increased dietary fiber, an overall increase in bacterial fermentation, reduction in intestinal luminal pH, and improvement in the solubilization of minerals such as Fe and Zn was observed in vivo [6,12,14]. Although we did not observe significant taxonomic shifts in the cecal microbiota of the animals receiving teff extract, the upregulation of many bacterial pathways relevant to Fe and Zn metabolism is a profound and novel finding. Additional studies are now warranted to assess cecal microbiota shifts post hatch and during a longer feeding trial.

## 5. Conclusions

The present study demonstrates that teff contains high amounts of fiber, phytic acid, and the polyphenols ferulic, caffeic, and protocatechuic acids, which have been shown to improve micronutrient absorption. Intra-amniotic administration of various concentrations of teff extract improved brush border membrane functionality through increases in villus architecture, surface area, and goblet cell expansion and related mucin production. Consequently, this contributed to increased relative expression of various duodenal enzymes responsible for mineral absorption and transport, and increased levels of hepatic Fe and Zn concentration, although serum concentrations remained unchanged. Although we did not observe significant alterations in the taxonomy of the cecal microbiota, various relevant bacterial metabolic pathways, such as fatty acid synthesis, were upregulated, which may demonstrate that teff administration positively influences the metagenome of the cecal microbiota, thus maximizing the solubilization and absorption of micronutrients in the gut. Given these findings, teff appears to represent a promising staple food crop and should be further evaluated in both long-term animal and controlled human efficacy trials. 

## Figures and Tables

**Table 1:** DNA Sequences of the primers used in this study.

Analyte	Forward Primer (5'→3')	Reverse Primer (5'→3')	Base Pair	GI Identifier
Calcium Metabolism				
TRPV6	GCTCCCAGAACCTTCTCTATTT	CCAGGTAATCCTGAGCTCTAATG	123	418307
PMCA1b	TGCAGATGCTGTGGGTAAAT	CCATAAGGCTTCCGCAATAGA	100	374244
NXC1	CCTGACGGAGAAATAAGGAAGA	CCCAGGAGAAGACACAGATAAA	114	395760
Iron Metabolism				
DMT1	TTGATTCAGAGCCTCCCATTAG	GCGAGGAGTAGGCTTGTATTT	101	206597489
Ferroportin	CTCAGCAATCACTGGCATCA	ACTGGGCAACTCCAGAAATAAG	98	61098365
DcytB	CATGTGCATTCTCTTCCAAAGTC	CTCCTTGGTGACCGCATTAT	103	20380692
Inflammatory Response				
IL-1β	CTCACAGTCCTTCGACATCTTC	TGTTGAGCCTCACTTTCTGG	119	88702685
IL-6	ACCTCATCCTCCGAGACTTTA	GCACTGAAACTCCTGGTCTT	105	302315692
TNF-α	GACAGCCTATGCCAACAAGTA	TTACAGGAAGGGCAACTCATC	109	53854909
Magnesium Metabolism				
MRS2	GCTGGTAACCGGGATTATGT	GCAGGAACATGAGGAGGTAAT	105	420820
TRPM6	ACAGATGCTGCTGACTGATATG	AAGATAGTGGGTGGTAGGAGAA	99	100859603
TRPM7	GCGTGGGATAGAGTTGACATT	TCACAAGGGCATCCAACATAG	100	427502
Zinc Metabolism				
ZnT1	GGTAACAGAGCTGCCTTAACT	GGTAACAGAGCTGCCTTAACT	105	54109718
ZnT7	GGAAGATGTCAGGATGGTTCA	CGAAGGACAAATTGAGGCAAAG	87	56555152
ZIP9	CTAAGCAAGAGCAGCAAAGAAG	CATGAACTGTGGCAACGTAAAG	100	237874618
Δ6 desaturase	GGCGAAAGTCAGCCTATTGA	AGGTGGGAAGATGAGGAAGA	93	261865208
Hypertension				
ACE	CATGGCCTTGTCTGTCTCC	GAGGTATCCAAAGGGCAGG	142	424059
AT1R	TCATCTGGCTCCTTGCTGG	AACCTAGCCCAACCCTCAG	138	396065
BBM Functionality				
AP	CGTCAGCCAGTTTGACTATGTA	CTCTCAAAGAAGCTGAGGATGG	138	45382360
SI	CCAGCAATGCCAGCATATTG	CGGTTTCTCCTTACCACTTCTT	95	2246388
SGLT1	GCATCCTTACTCTGTGGTACTG	TATCCGCACATCACACATCC	106	8346783
18s rRNA	GCAAGACGAACTAAAGCGAAAG	TCGGAACTACGACGGTATCT	100	7262899

**Table 2:** Concentrations of calcium, iron, magnesium, and zinc in teff flour and teff extract ^1^.

Treatment Group	Calcium (µg/g)	Iron (µg/g)	Magnesium (µg/g)	Zinc (µg/g)
**Teff seed**	2259.32 ± 24.73 ^a^	60.64 ± 0.92 ^b^	2023.49 ± 18.93 ^a^	42.87 ± 0.42 ^b^
**Teff seed extract**	2052.07 ± 123.95 ^b^	128.23 ± 11.60 ^a^	1088.87 ± 42.24 ^b^	53.84 ± 4.50 ^a^

^1^ Values are means ± SEM, *n* = 5. ^a,b^ Treatment groups not indicated by the same letter are significantly different (*p* < 0.05).

**Table 3:** Dietary fiber, protein, phytic acid, and phytate:Fe ratio in teff flour and teff extract ^1^.

Treatment Group	Insoluble Fiber (g/100 g)	Soluble Fiber (g/100 g)	Total Fiber (g/100 g)	Protein (g/100 g)	Phytic Acid (g/100 g)	Phytic Acid:Iron Ratio
**Teff seed**	8.32 ± 0.23 ^a^	2.10 ± 0.12 ^b^	10.43 ± 0.11 ^b^	10.02± 0.39 ^a^	0.14± 0.02 ^a^	1.74 ± 0.03 ^a^
**Teff seed extract**	6.15 ± 0.61 ^b^	6.98 ± 0.91 ^a^	13.13 ± 0.30 ^a^	10.86± 0.88 ^a^	0.17± 0.11 ^a^	0.41 ± 0.05 ^b^

^1^ Values are means ± SEM, *n* = 5. ^a,b^ Treatment groups not indicated by the same letter are significantly different (*p* < 0.05).

**Table 4:** Concentration of the five most common polyphenolic compounds in teff samples ^1^.

Polyphenolic Compounds	Mass (Da)	(M + H) (Da)	(M − H) (Da)	Retention Time (min)	Found in (MS mode)	
					Teff Seed (extract)	Teff Seed (flour)
Protocatechuic Acid	154.12	155.128	153.112	1.621	NEG	NEG
Caffeic Acid	180.16	181.168	179.152	2.958	POS/NEG	ND
Vanillic Acid	168.15	169.158	167.142	3.094	POS	ND
p- Coumaric Acid	164.16	165.168	164.152	3.895	POS/NEG	ND
Ferulic Acid	194.18	195.188	193.172	4.428	POS/NEG	POS

^1^ Values are means ± SEM, *n* = 5. MAU: milli absorbance unit; min: minutes. Da: Dalton; M + H: Mass + Hydrogen; M − H: Mass − Hydrogen; MS: Mass Spectrometry; ND: Not Determined; POS: Positive. NEG: Negative.

**Table 5:** Hemoglobin, body weight, cecum weight, and cecum:body weight ratio in all groups ^1^.

Treatment Group	Hemoglobin (g/dL)	Body Weight Average (g)	Cecum Weight Average (g)	Cecum:Body Weight Ratio
NI	8.747 ± 0.797 ^a^	45.4 ± 1.3 ^b^	0.5 ± 0.1 ^b^	0.010 ± 0.002 ^b^
18Ω H_2_O	10.603 ± 0.591 ^a^	46.9 ± 1.2 ^a^	0.5 ± 0.1 ^a^	0.011 ± 0.001 ^a^
5% Inulin	8.860 ± 0.690 ^a^	49.7 ± 0.9 ^a^	0.6 ± 0.1 ^a^	0.012 ± 0.001 ^a^
1% Teff	9.575 ± 1.138 ^a^	47.0 ± 1.0 ^a^	0.6 ± 0.1 ^a^	0.012 ± 0.001 ^a^
2.5% Teff	8.410 ± 0.920 ^a^	47.1 ± 1.1 ^a^	0.5 ± 0.0 ^b^	0.010 ± 0.001 ^b^
5% Teff	8.501 ± 0.874 ^a^	46.5 ± 1.3 ^a^	0.7 ± 0.1 ^a^	0.015 ± 0.002 ^a^
7.5% Teff	9.569 ± 0.633 ^a^	47.4 ± 1.4 ^a^	0.6 ± 0.0 ^a^	0.012 ± 0.001 ^a^

^1^ Values are means ± SEM, *n* = 8. ^a,b^ Treatment groups not indicated by the same letter are significantly different (*p* < 0.05). NI = non-injected.

**Table 6:** Fe and Zn concentrations in liver and serum ^1^.

Treatment Groups	Liver		Serum	
	Iron (µg/g)	Zinc (µg/g)	Iron (µg/g)	Zinc (µg/g)
NI	32.47 ± 2.83 ^b^	15.79 ± 0.95 ^c^	2.09 ± 0.24 ^a^	0.86 ± 0.08 ^a^
18Ω H_2_O	37.93 ± 4.93 ^ab^	16.12 ± 0.96 ^c^	2.00 ± 0.27 ^a^	0.84 ± 0.07 ^a^
5% Inulin	48.96 ± 4.39 ^a^	18.23 ± 0.88 ^abc^	2.76 ± 0.33 ^a^	0.97 ± 0.08 ^a^
1% Teff	37.40 ± 3.67 ^ab^	17.61 ± 0.91 ^bc^	2.22 ± 0.40 ^a^	0.91 ± 0.08 ^a^
2.5% Teff	36.39 ± 3.54 ^ab^	22.82 ± 2.42 ^ab^	2.09 ± 0.22 ^a^	0.89 ± 0.06 ^a^
5% Teff	39.40 ± 3.89 ^ab^	23.37 ± 3.10 ^ab^	2.83 ± 0.28 ^a^	0.81 ± 0.07 ^a^
7.5% Teff	46.44 ± 5.83 ^a^	24.14 ± 3.33 ^a^	2.88 ± 0.38 ^a^	1.02 ± 0.07 ^a^

^1^ Values are means ± SEM, *n* = 8. ^a,b,c^ Treatment groups not indicated by the same letter are significantly different (*p* < 0.05). NI = non-injected.

**Table 7:** Glycogen and liver ferritin concentrations ^1^.

Treatment Group	Glycogen (mg/g)	Liver Ferritin (AU)
NI	0.034 ± 0.011 ^a^	1.974 ± 0.005 ^a^
18Ω H_2_O	0.022 ± 0.003 ^a^	1.534 ± 0.519 ^a^
5% Inulin	0.029 ± 0.005 ^a^	0.148 ± 0.005 ^b^
1% Teff	0.035 ± 0.006 ^a^	1.491 ± 0.299 ^a^
2.5% Teff	0.022 ± 0.007 ^a^	0.275 ± 0.245 ^b^
5% Teff	0.044 ± 0.017 ^a^	0.031± 0.001 ^b^
7.5% Teff	0.021 ± 0.007 ^a^	0.034 ± 0.001 ^b^

^1^ Values are means ± SEM, *n* = 8. ^a,b^ Treatment groups not indicated by the same letter are significantly different (*p* < 0.05). NI = non-injected.

**Table 8:** Effect of the intra-amniotic administration of experimental teff solutions on duodenal villus and crypts measurements ^1^.

Treatment Group	Villus Length (µm)	Villus Diameter (µm)	Depth of Crypts (µm)
NI	223.29 ± 3.44 ^ab^	53.15 ± 0.73 ^b^	66.30 ± 1.33 ^b^
18Ω H_2_O	224.14 ± 4.10 ^ab^	46.84 ± 0.69 ^c^	53.42 ± 1.11 ^c^
5% Inulin	274.11 ± 3.92 ^b^	43.66 ± 0.64 ^d^	51.30 ± 1.06 ^c^
1% Teff	266.85 ± 4.39 ^a^	56.22 ± 0.78 ^a^	73.62 ± 1.40 ^a^
2.5% Teff	263.66 ± 3.85 ^a^	53.44 ± 0.75 ^ab^	65.74 ± 1.26 ^b^
5% Teff	302.83 ± 2.95 ^ab^	54.80 ± 0.67 ^ab^	67.75 ± 1.43 ^b^
7.5% Teff	290.86 ± 3.12 ^a^	50.00 ± 0.02 ^ab^	66.38 ± 1.25 ^b^

^1^ Values are the means ± SEM, *n* = 5. a–d Treatment groups not indicated by the same letter are significantly different (*p* < 0.05). NI = non-injected.

**Table 9:** Effect of the intra-amniotic administration of experimental teff solutions on duodenal goblet cells ^1^.

Treatment Group	Goblet Cell Diameter (µM)	Crypts Goblet Cell Number	Total Villus Goblet Cell Number	Villus Goblet Cell Number		
				Acidic	Neutral	Mixed
NI	6.50 ± 0.06 ^cd^	9.53 ± 0.32 ^b^	51.32 ± 1.34 ^b^	10.22 ± 0.26 ^a^	0.12 ± 0.03 ^a^	0.13 ± 0.03 ^c^
18Ω H_2_O	6.37 ± 0.06 ^de^	10.01 ± 0.22 ^b^	44.83 ± 1.16 ^c^	9.46 ± 0.23 ^b^	0.00 ± 0.00 ^b^	0.05 ± 0.03 ^d^
5% Inulin	6.06 ± 0.04 ^f^	12.27 ± 0.33 ^a^	54.56 ± 1.40 ^b^	10.65 ± 0.25 ^a^	0.00 ± 0.00 ^b^	0.02 ± 0.01 ^d^
1% Teff	6.82 ± 0.05 ^b^	12.15 ± 0.28 ^a^	61.76 ± 1.56 ^a^	9.37 ± 0.24 ^b^	0.00 ± 0.00 ^b^	0.22 ± 0.03 ^b^
2.5% Teff	7.54 ± 0.05 ^a^	11.81 ± 0.24 ^a^	54.27 ± 1.20 ^b^	6.21 ± 0.15 ^d^	0.00 ± 0.00 ^b^	0.32 ± 0.04 ^a^
5% Teff	6.29 ± 0.06 ^e^	10.11 ± 0.27 ^b^	42.54 ± 0.93 ^c^	8.33 ± 0.20 ^c^	0.00 ± 0.00 ^b^	0.18 ± 0.03 ^bc^
7.5% Teff	6.64 ± 0.06 ^bc^	9.51 ± 0.24 ^b^	43.12 ± 0.98 ^c^	7.81 ± 0.19 ^c^	0.00 ± 0.00 ^b^	0.16 ± 0.03 ^bc^

^1^ Values are the means ± SEM, *n* = 5. a–f Treatment groups not indicated by the same letter are significantly different (*p* < 0.05). NI = non-injected.

## Supplementary Materials

The following are available online at https://www.mdpi.com/2072-6643/12/10/3020/s1.
