# Supplementary material for: Alterations in the Intestinal Morphology, Gut Microbiota, and Trace Mineral Status Following Intra-Amniotic Administration (Gallus gallus) of Teff (Eragrostis tef) Seed Extracts
Source: Nutrients. 2020 Oct 2;12(10):3020. doi: 10.3390/nu12103020 (PMC7601863; doi:10.3390/nu12103020)
Supplement: Supplementary file 1 [file nutrients-12-03020-s001.pdf]

Supplementary Materials

Alterations in the Intestinal Morphology, Gut Microbiota, and Trace Mineral Status Following Intra-Amniotic Administration (*Gallus gallus*) of Teff (*Eragrostis tef*) Seed Extracts

Johnathon Carboni, Spenser Reed, Nikolai Kolba, Adi Eshel, Omry Koren and Elad Tako

1. Materials and Methods

2.5. Protein and Fiber Analysis

Total nitrogen concentrations were measured in a 500 mg sample of teff flour or teff extract by the Dumas combustion method at A&L Great Lakes Laboratories (Fort Wayne, IN, USA) in accordance with AOAC method 968.06 [AOAC *Official Methods of Analysis of AOAC International*; 17th ed.; Association of Official Analytical Chemists: Rockville, MD, USA, 2000]. The percentage of crude protein was estimated by multiplying the dry weight total nitrogen concentration by a factor of 6.25 [Jones, D. *Factors for converting percentages of nitrogen in foods and feeds into percentages of proteins*; USDA: Washington D.C., 1931]. Insoluble, soluble and total fiber concentrations were determined by the enzymatic-gravimetric AOAC method 985.29 [AOAC *Official Methods of Analysis of AOAC International*; 16th ed.; Association of Official Analytical Chemists International: Gaithersburg, MD, USA, 1997; Vol. 2], using enzymatic hydrolysis with heat-resistant amylase, protease and amyloglucosidase (Total Dietary Fiber Assay Kit, Sigma Aldrich Co., St. Louis, MO, USA).

2.6.1. Liquid Chromatography-Mass Spectroscopy (LC-MS) Analysis of Polyphenols

Extracts and standards were analyzed by an Agilent 1220 Infinity Liquid Chromatograph (LC; Agilent Technologies, Inc., Santa Clara, CA, USA) coupled to an Advion expressionL® compact mass spectrometer (CMS; Advion Inc., Ithaca, NY, USA). Two-μL samples were injected and passed through an Acquity™ UPLC BEH Shield RP18 1.7 μm 2.1 x 100 mm column (Waters, Milford, MA, USA) at 0.35 mL/minute. The column was temperature-controlled at 45°C. The mobile phase consisted of ultra-pure water with 0.10 % formic acid (solvent A) and acetonitrile with 0.10 % formic acid (solvent B). Polyphenols were eluted using linear gradients of 86.7 to 77.0% A in 0.50 minutes, 77.0 to 46.0% A in 5.50 minutes, 46.0 to 0% A in 0.50 minutes, hold at 0% A for 3.50 minutes, 0 to 86.7% A in 0.50 minutes, and hold at 86.7% A for 3.50 minutes for a total run time of 14 minutes. From the column, flow was directed into a variable wavelength UV detector set at 254, 278, 295 nm. Flow was then directed into the source of an Advion expressionL® CMS, and electro spray ionization (ESI) mass spectrometry was performed in negative ionization mode using selected ion monitoring with a scan time of 50 milliseconds for the 3 polyphenol masses of interest. Capillary temperature and voltages were 300°C and 100 volts, respectively. ESI source voltage and gas temperature were 2.6 kilovolts and 240°C respectively. Desolvation gas flow was 240 L/hour. Advion Mass Express™ software was used to control the LC and CMS instrumentation and data acquisition. Individual polyphenols were identified and confirmed by comparison of m/z and LC retention times with authentic standards. Polyphenol standard curves for flavonoids were derived from integrated areas under UV absorption peaks from 5 replications. Standard curves for caffeic acid, ferulic, protocatechuic acids were constructed from MS ion intensities using 5 replications.

2.9. Isolation of Total RNA from Chicken Duodenum and Liver

Total RNA was extracted from 30 mg of the proximal duodenal tissue or liver tissue (n = 8) using Qiagen RNeasy Mini Kit (RNeasy Mini Kit, Qiagen Inc., Valencia, CA, USA) according to the manufacturer's protocol. Briefly, tissues were disrupted and homogenized with a rotor-stator homogenizer in buffer RLT®, containing β-mercaptoethanol. The tissue lysate was centrifuged for 3 minutes at 8,000 g in a micro centrifuge. An aliquot of the supernatant was transferred to another tube, combined with 1 volume of 70% ethanol and mixed immediately. Each sample (700 μL) was applied to an RNeasy mini column, centrifuged for 15 s at 8000 g, and the flow through material was discarded. Next, the RN easy columns were transferred to new 2-mL collection tubes, and 500 μL of buffer RPE® was pipetted onto the RNeasy column followed by centrifugation for 15 s at 8000 g. An additional 500 μL of buffer RPE were pipetted onto the RNeasy column and centrifuged for 2 min at 8000 g. Total RNA was eluted in 50 μL of RNase free water. All steps were carried out under RNase free conditions. RNA was quantified by absorbance at A 260/280. Integrity of the 18S ribosomal RNAs was verified by 1.5% agarose gel electrophoresis followed by ethidium bromide staining. DNA contamination was removed using TURBO DNase treatment and removal kit from AMBION (Austin, TX, USA).

2.10. Real Time Polymerase Chain Reaction (RT-PCR)

To create the cDNA, a 20 μL reverse transcriptase (RT) reaction was completed in a BioRad C1000 touch thermocycler using the Improm-II Reverse Transcriptase Kit (Catalog #A1250; Promega, Madison, WI, USA). The first step consisted of 1 μg of total RNA template, 10 μMof random hexamer primers, and 2 mM of oligo-dT primers. The RT protocol was to anneal primers to RNA at 94°C for 5 min, copy the first strand for 60 min at 42°C (optimum temperature for the enzyme), then heat inactivate at 70°C for 15 min and hold at 4°C until ready to analyze by Nanodrop (Waltham, MA, USA). The concentration of cDNA obtained was determined by measuring the absorbance at 260 nm and 280 nm using an extinction coefficient of 33 (for single stranded DNA). Genomic DNA contamination was assessed by a real-time RT-PCR assay for the reference genes samples.

2.10.1. Primer Design

The primers used in the real-time qPCR was designed based on 22 gene sequences from Genbank database, using Real-Time Primer Design Tool software (IDT DNA, Coralvilla, IA, USA). The sequences and the description of the primers used in this work are summarized in Table 1. The amplicon length was limited to 90 to 150 bp. The length of the primers was 17–25-mer and the GC content was between 41% and 55%. The specificity of the primers was tested by performing a BLAST search against the genomic National Center for Biotechnology Information (NCBI) database. The *Gallus gallus* primer 18S rRNA was designed as a reference gene. Results obtained from the qPCR system were used to normalize those obtained from the specific systems.

2.10.2. Real-Time qPCR Design

cDNA was used for each 10 μL reaction together with 2×BioRad SSO Advanced Universal SYBR Green Supermix (Cat #1725274, Hercules, CA, USA) which included buffer, Taq DNA polymerase, dNTPs and SYBR green dye. Specific primers (forward and reverse) (Table 1) and cDNA or water (for no template control) were added to each PCR reaction. The specific primers used can be seen in Table 1. For each gene, the optimal $MgCl_2$ concentration produced the amplification plot with the lowest cycle product (Cp), the highest fluorescence intensity and the steepest amplification slope. Master mix (8 μL) was pipetted into the 96-well plate and 2 μL cDNA was added as PCR template. Each run contained 7 standard curve points in duplicate. A no template control of nuclease-free water was included to exclude DNA contamination in the PCR mix. The double stranded DNA was amplified in the Bio-Rad CFX96 Touch (Hercules, CA, USA) using the following PCR conditions: initial denaturing at 95°C for 30 s, 40 cycles of denaturing at 95°C for 15 s, various annealing temperatures according to Integrated DNA Technologies (IDT) for 30 s and elongating at 60°C for 30 s.

The data on the expression levels of the genes were obtained as Cp values based on the "second derivative maximum" (automated method) as computed by Bio-Rad CFX Maestro 1.1 (Version 4.1.2433.1219, Hercules, CA, USA). For each of the 13 genes, the reactions were run in duplicate. All assays were quantified by including a standard curve in the real-time qPCR analysis. The next four points of the standard curve were prepared by a 1:10 dilution. Each point of the standard curve was included in duplicate. A graph of Cp vs. log 10 concentrations was produced by the software and the efficiencies were calculated as 10[1/slope]. The specificity of the amplified real-time RT-PCR products were verified by melting curve analysis (60–95°C) after 40 cycles, which should result in a number of different specific products, each with a specific melting temperature.

2.11. Collection of Microbial Samples and Intestinal Contents DNA Isolation

The contents of the cecum were placed into a sterile 15 mL tube containing 9 mL of sterile PBS and homogenized by vortexing with glass beads (3 mm diameter) for 3 min. Debris was removed by centrifugation at 1000 g for 5 min, and the supernatant was collected and centrifuged at 4,000 ×g for 10 min. The pellet was washed with PBS and stored at -20°C until DNA extraction. For DNA purification, the pellet was re-suspended in 50 mM EDTA and treated with lysozyme (Sigma Aldrich CO., St. Louis, MO, USA; final concentration of 10 mg/mL) for 60 min at 37°C. The bacterial genomic DNA was isolated using a Wizard Genomic DNA purification kit (Promega Corp., Madison, WI, USA).

2.12. Glycogen Analysis

At hatch, the pectorals muscle (20 mg) was dissected and placed on ice before freezing for subsequent glycogen analysis. The pectoral muscle samples were then homogenized in 8% perchloric acid, and glycogen content was determined using modified methods described by Dreiling et al. [1]. After homogenization, the samples were centrifuged at 12,000 rpm at 4°C for 15 min. The supernatant was removed, and 1.0 mL of petroleum ether was added to each tube. After mixing, the petroleum ether fraction was removed, and samples from the bottom layer were transferred to a new tube containing 300 μL of color reagent. All samples were read at a wavelength of 450 nm in ELISA reader and the amount of glycogen was calculated according to a standard curve. The amount of glycogen present in pectoral sample was determined by multiplying the weight of the tissue by the amount of glycogen per 1 g of wet tissue.

2.13. Liver Ferritin Analysis

As previously described, [2-4], 1 g of sample was diluted into 1 mL of 50 mM Hepes buffer, pH 7.4, and homogenized on ice for 2 min (5000 g). One mL of each homogenate was subjected to heat treatment for 10 min at 75°C to aid isolation of ferritin (other proteins are not stable at that temperature). Subsequently, samples were immediately cooled down on ice for 30 min. Thereafter, samples were centrifuged for 30 min (13000 g) at 4°C until a clear supernatant was obtained and the pellet containing most of the insoluble denatured proteins was discarded. Native polyacrylamide gel electrophoresis was conducted using a 6% separating gel and a 5% stacking gel. Samples were run at a constant voltage of 100 V. Thereafter, gels were treated with either of the two stains: Coomasie blue G-250 stain, specific for proteins, or potassium ferricyanide (K3Fe (CN)6) stain, specific for Fe. The corresponding band found in the protein and Fe stained gel was considered to be ferritin. Measurements of the bands were conducted using the Quantity-One-1-D analysis program (Bio-Rad, Hercules, CA). The local background was subtracted from each sample. Horse spleen ferritin (Sigma Aldrich Co., St. Louis, MO) was used as a standard for calibrating ferritin protein and Fe concentrations of the samples. Dilutions of the horse spleen ferritin were made and treated similarly to the liver supernatant samples in order to create a reference line for both protein and Fe stained gels.

2.14. Morphological Examination

Intestine and pectoral samples were collected at the conclusion of the study and from each treatment group (n = 5 per group). Samples were fixed in fresh 4% (v/v) buffered formaldehyde, dehydrated, cleared, and embedded in paraffin. Serial sections were cut at 5 μm and placed on glass slides. Intestinal sections were deparaffinized in xylene, rehydrated in a graded alcohol series, stained with Alcian Blue/Periodic acid-Schiff, and examined by light microscopy. The following variables were measured in the intestine: villus height, villus width, depth of crypts, paneth cells, goblet cell number, goblet cell diameter, types of goblet cells in the villi epithelium, goblet cells within the crypts and the mucus layer thickness in each segment were performed with a light microscope using EPIX XCAP software (Standard version, Olympus, Waltham, MA, USA). There were 4 segments for each biological sample and five biological samples per treatment group. Goblet cells were enumerated on 10 villi/sample, and the means were utilized for statistical analysis.

The pectoral samples were stained with Hematoxylin-Eosin and examined by light microscopy. The following variables were measured in the pectoral samples: muscle fascicle diameter, muscle fiber number and diameter, and satellite cell number performed with the same light microscope and EPIX XCAP software. Satellite cells were enumerated per muscle fascicle. There were 4 samples per biological sample and five biological samples per treatment group.

2.15. 16S rRNA Gene Amplification, Sequencing, and Analysis

As previously described by Dias *et* al. (2018), microbial genomic DNA was extracted from cecal samples using the PowerSoil DNA isolation kit, as described by the manufacturer (MoBio Laboratories Ltd., Carlsbad, CA, USA). Bacterial 16S rRNA gene sequences were PCR-amplified from each sample using the 515F-806R primers for the V4 hypervariable region of the 16S rRNA gene, including 12-base barcodes, as previously published [5]. PCR procedure reactions consisted of 25 μL Primestar max PCR mix (Takara Kusatsu, Shiga, Japan), 2 μM of each primer, 17 μL of ultra-pure water, and 4 μL DNA template. Reaction conditions consisted of an initial denaturing step for 3 min at 95°C followed by 30 cycles of 10s at 98°C, 5s at 55°C, 20s at 72°C, and final elongation at 72°C for 1 min. PCR products were then purified with Ampure magnetic purification beads (Beckman Coulter, Atlanta, GA, USA) and quantified using a Quant-iT PicoGreen dsDNA quantitation kit (Invitrogen, Carlsbad, CA, USA). Equimolar ratios of total samples were pooled and sequenced at the Azrieli Faculty of Medicine of the Bar Ilan University (Safed, Israel) using an Illumina MiSeq Sequencer (Illumina, Inc., Madison, WI, USA).

As previously described [6], data analysis was performed using QIIME2 [7]. Sequence reads were demultiplexed by per-sample barcodes and Illumina-sequenced amplicon reads errors were corrected by Divisive Amplicon Denoising Algorithm (DADA2) [8]. A phylogenetic tree was generated and sequences were classified taxonomically using the Greengenes [9] reference database at a confidence threshold of 99%. The Greengenes taxonomies were used to generate summaries of the taxonomic distributions of features across different levels (phylum, order, family, and genus). After filtration of low abundant features (observed in less than 2 samples per group), alpha and beta diversity analysis were calculated based on a feature table containing at least 9800 sequences per sample. Microbial richness, an alpha diversity parameter, was calculated using Faith's Phylogenetic Diversity [10]. Beta diversity was analyzed using Jaccard similarity distances [11]. Linear discriminant analysis Effect Size (LEfSe) [12] was used to determine the features significantly differ between samples according to relative abundances. Metagenome functional predictive analysis was carried out using phylogenetic investigation of communities by reconstruction of unobserved states (PICRUSt) [13] software (version 1.1.3). Briefly, feature abundance was normalized by 16S rRNA gene copy number, identified and compared to a phylogenetic reference tree using the Greengenes database, and was assigned functional traits and abundance based on known genomes and prediction using the Kyoto Encyclopedia of Genes and Genomes (KEGG). Data representing significant fold-change differences in functional pathways between experimental groups was plotted.

Supplementary References:

1. 1. Dreiling, C.E.; Brown, D.E.; Casale, L.; Kelly, L. Muscle glycogen: Comparison of iodine binding and enzyme digestion assays and application to meat samples. *Meat Sci.* **1987**, *20*, 167–177.
2. 2. Mete, A.; Van Zeeland, Y.R.; Vaandrager, A.B.; van Dijk, J.E.; Marx, J.J.; Dorrestein, G.M.; Partial purification and characterization of ferritin from the liver and intestinal mucosa of chickens, turtledoves and mynahs. *Avian Pathol*. **2005**, 34(5), 430–434.
3. 3. Passaniti, A.; Roth,T.F. Purification of chicken liver ferritin by two novel methods and structural comparison with horse spleen ferritin. *J. Biochem.* **1989**, 258, 413–419.
4. 4. Tako, E.; Rutzke, M.A.; Glahn, R.P. Using the domestic chicken (Gallus gallus) as an in vivo model for iron bioavailability1. *Poultry Science* **2010**, *89*, 514–521.
5. 5. Caporaso, J.G.; Lauber, C.L.; Walters, W.A.; Berg-Lyons, D.; Huntley, J.; Fierer, N.; Owens, S.M.; Betley, J.Fraser, L.; Bauer, M.; et al. Ultra-high-throughput microbial community analysis on the Illumina HiSeq and MiSeq platforms. *ISME J.* **2012**, 6, 1621–1624.
6. 6. Dias, D.M.; Kolba, N.; Hart, J.J.; Ma, M.; Sha, S.T.; Lakshmanan, N.; Nutti, M.R.; Martino, H.S.D.; Glahn, R.P.; Tako, E. Soluble extracts from carioca beans (Phaseolus vulgaris L.) affect the gut microbiota and iron related brush border membrane protein expression in vivo (Gallus gallus). *Food Res. Int.* **2019**, *123*, 172–180.
7. 7. Caporaso, G.J.; Kuczynski, J.; Stombaugh, J.; Bittinger, K.; Bushman, F.D.; Costello, E.K.; Fierer, N.; Peña, A.G.; Goodrich, J.K.; Gordon, J.I.; et al. Correspondence QIIME allows analysis of high- throughput community sequencing data Intensity normalization improves color calling in SOLiD sequencing. *Nat. Publ. Gr.* **2010**, 7, 335–336. https://doi.org/10.1038/s41587-019-0209-9
8. 8. Callahan, B.J.; McMurdie, P.J.; Rosen, M.J.; Han, A.W.; Johnson, A.J.A.; Holmes, S.P. High-resolution sample inference from Illumina amplicon data. *Nat. Methods.* **2016**, 13, 581–583.
9. 9. DeSantis, T.Z.; Hugenholtz, P.; Larsen, N.; Rojas, M.; Brodie, E.L.; Keller, K.; Huber, T.; Dalevi, D.; Hu, P.; Andersen, G.L. Greengenes, a chimera-checked 16S rRNA gene database and workbench compatible with ARB. *Appl. Environ. Microbiol.* **2006**, 72, 5069–5072.
10. 10. Faith, D.P. Conservation evaluation and phylogentic diversity. *Biol. Conserv.* **1992**, 61, 1–10.
11. 11. Chao, A.; Chiu, C.H.; Hsieh. T.C. Proposing a resolution to debates on diversity partitioning. *Ecology*. **2012**,93(9):2037-51; doi: 10.1890/11-1817.1.
12. 12. Segata, N.; Izard, J.;Waldron, L.; Gevers, D.;Miropolsky, L.; Garrett,W.S.; Huttenhower, C. LEfSe-Metagenomic biomarker discovery and explanation. *Genome Biol*. **2011**, 12, R60.
13. 13. Langille, M.G.I.; Zaneveld, J.; Caporaso, J.G.; McDonald, D.; Knights, D.; Reyes, J.A.; Clemente, J.C.; Burkepile, D.E.; Thurber, R.L.V.; Knight, R.; et al. Predictive functional profiling of microbial communities using 16S rRNA marker gene sequences. *Nat. Biotechnol*. **2013**, 31, 814–821.
